# The pathobiology of psychomotor slowing in psychosis: altered cortical excitability and connectivity

**DOI:** 10.1093/brain/awad395

**Published:** 2023-11-20

**Authors:** Stephanie Lefebvre, Gwendolyn Gehrig, Niluja Nadesalingam, Melanie G Nuoffer, Alexandra Kyrou, Florian Wüthrich, Sebastian Walther

**Affiliations:** Translational Research Center, University Hospital of Psychiatry and Psychotherapy, University of Bern, 3000 Bern, Switzerland; Translational Imaging Center (TIC), Swiss Institute for Translational and Entrepreneurial Medicine, 3000 Bern, Switzerland; Translational Research Center, University Hospital of Psychiatry and Psychotherapy, University of Bern, 3000 Bern, Switzerland; Translational Research Center, University Hospital of Psychiatry and Psychotherapy, University of Bern, 3000 Bern, Switzerland; Translational Imaging Center (TIC), Swiss Institute for Translational and Entrepreneurial Medicine, 3000 Bern, Switzerland; Translational Research Center, University Hospital of Psychiatry and Psychotherapy, University of Bern, 3000 Bern, Switzerland; Translational Imaging Center (TIC), Swiss Institute for Translational and Entrepreneurial Medicine, 3000 Bern, Switzerland; Graduate School for Health Sciences, University of Bern, 3000 Bern, Switzerland; Translational Research Center, University Hospital of Psychiatry and Psychotherapy, University of Bern, 3000 Bern, Switzerland; Translational Research Center, University Hospital of Psychiatry and Psychotherapy, University of Bern, 3000 Bern, Switzerland; Translational Imaging Center (TIC), Swiss Institute for Translational and Entrepreneurial Medicine, 3000 Bern, Switzerland; Translational Research Center, University Hospital of Psychiatry and Psychotherapy, University of Bern, 3000 Bern, Switzerland; Translational Imaging Center (TIC), Swiss Institute for Translational and Entrepreneurial Medicine, 3000 Bern, Switzerland

**Keywords:** motor inhibition, SICI, catatonia, resting-state fMRI, diffusion imaging

## Abstract

Psychomotor slowing is a frequent symptom of schizophrenia. Short-interval intracortical inhibition assessed by transcranial magnetic stimulation demonstrated inhibitory dysfunction in schizophrenia. The inhibitory deficit results from additional noise during information processing in the motor system in psychosis. Here, we tested whether cortical inhibitory dysfunction was linked to psychomotor slowing and motor network alterations.

In this cross-sectional study, we included 60 patients with schizophrenia and psychomotor slowing determined by the Salpêtrière Retardation Rating Scale, 23 patients without slowing and 40 healthy control participants. We acquired single and double-pulse transcranial magnetic stimulation effects from the left primary motor cortex, resting-state functional connectivity and diffusion imaging on the same day. Groups were compared on resting motor threshold, amplitude of the motor evoked potentials, as well as short-interval intracortical inhibition. Regression analyses calculated the association between motor evoked potential amplitudes or cortical inhibition with seed-based resting-state functional connectivity from the left primary motor cortex and fractional anisotropy at whole brain level and within major motor tracts.

In patients with schizophrenia and psychomotor slowing, we observed lower amplitudes of motor evoked potentials, while the short-interval intracortical inhibition/motor evoked potentials amplitude ratio was higher than in healthy controls, suggesting lower cortical inhibition in these patients. Patients without slowing also had lower amplitudes of motor evoked potentials. Across the combined patient sample, cortical inhibition deficits were linked to more motor coordination impairments. In patients with schizophrenia and psychomotor slowing, lower amplitudes of motor evoked potentials were associated with lower fractional anisotropy in motor tracts. Moreover, resting-state functional connectivity between the primary motor cortex, the anterior cingulate cortex and the cerebellum increased with stronger cortical inhibition. In contrast, in healthy controls and patients without slowing, stronger cortical inhibition was linked to lower resting-state functional connectivity between the left primary motor cortex and premotor or parietal cortices.

Psychomotor slowing in psychosis is linked to less cortical inhibition and aberrant functional connectivity of the primary motor cortex. Higher neural noise in the motor system may drive psychomotor slowing and thus may become a treatment target.

## Teaser

Lefebvre *et al.* demonstrate that psychomotor slowing in psychosis is linked to less cortical inhibition and aberrant functional connectivity of the primary motor cortex. They conclude that higher levels of neural noise in the motor system may drive psychomotor slowing and could represent a potential treatment target.

## Introduction

Schizophrenia is a devastating disorder, characterized by eight symptom dimensions, i.e. delusions, hallucinations, disorganization, negative symptoms, abnormal psychomotor behaviour, impaired cognition, depression and mania.^1^ Symptoms are thought to arise from aberrant brain connectivity at multiple levels.^2,3^

Abnormal psychomotor behaviour may include a general slowing of fine and gross motor behaviour, i.e. psychomotor slowing, but also specific motor phenomena such as dyskinesia, parkinsonism or catatonia.^4-6^ Motor abnormalities can be observed in chronic patients, but also in treatment-naïve first episode psychosis patients or even in subjects at risk for psychosis.^5,7-11^ Finally, motor abnormalities severely impair social and community functioning and predict poor clinical outcome.^12-14^

Psychomotor slowing is frequent in schizophrenia and thought to arise from poor movement preparation, altered motor coordination, impaired movement execution as well as complex interactions with altered emotion processing.^15-23,24^ The phenomenon can be assessed with clinical rating scales, instrumental measures such as actigraphy, or neurocognitive tests.^6,15^ Within schizophrenia, we found a subgroup with severe psychomotor slowing that presented with reduced physical activity, slower gait and compromised dexterity.^24^ Thus, a proportion of psychosis patients presents with severely altered behaviour, pointing to distinct pathobiology in subjects with psychomotor slowing.

Neuroimaging studies suggest multiple alterations in the motor circuitry in schizophrenia in general,^25^ including reduced grey matter in premotor areas, higher structural connectivity between cortical and subcortical motor areas,^26^ or higher resting-state functional connectivity (rsFC) between premotor/motor cortices and basal ganglia, thalamus, as well as cerebellum.^27-29^ In addition, specific alterations in the motor circuit were linked to hypokinetic motor abnormalities in psychosis.^4,25,30^ For example, patients with psychomotor slowing in the context of catatonia had higher resting-state cerebral blood flow in the supplementary motor area (SMA), higher fractional anisotropy (FA) in the left cortico-spinal tract, higher thalamo-cortical rsFC, and less flexible shifts in resting-state networks.^27,31-36^ However, the direction of rsFC changes between thalamus and primary motor cortex (M1) is debated. While some linked motor inhibition and negative symptoms to thalamocortical hyperconnectivity,^27,37^ others reported lower connectivity with psychomotor slowing,^38^ triggering the assumption that psychomotor slowing was linked to hypoconnectivity in the motor circuit.^39^

Currently, the field still struggles to relate the observed neural alterations in psychosis to pathophysiology or specific symptoms. Particularly, the physiology of the motor and premotor cortices remains unknown. Higher neural activity or higher rsFC in the premotor and motor cortices may arise from either too much excitatory action or too little inhibitory feedback. One key feature of schizophrenia pathobiology is noisy signalling in the cortex as evidenced by the excitation-inhibition imbalance, particularly in the frontal cortex, which is thought to result from impaired inhibitory action.^40^ In fact, reduced cortical inhibition seemed to be associated with more noise in the motor system.^41-44^ Indeed, post-mortem studies have substantiated reduced GABAergic tone in schizophrenia.^45^ For example, reduced GABA-related mRNA expression was detected in multiple cortex regions, including M1.^46^ In addition, *in vivo* spectroscopy studies indicated reduced levels of GABA in schizophrenia,^45^ while a SPECT study suggested reduced GABA receptor density in M1 in catatonia.^47^

Single and double pulse transcranial magnetic stimulation (TMS) experiments allow testing M1 physiology.^48^ The amplitude of motor evoked potential (MEP) informs on the excitability of corticospinal neurons and the influence of GABAergic and glutamatergic tone.^49^ The short-interval intracortical inhibition (SICI) paradigm applies two stimuli over M1, where the first subthreshold stimulus triggers a reduced response to a subsequent stimulus. SICI resembles another indirect *in vivo* probe of GABA_A_-related activity.^45,49^ Higher cortical inhibition seems to be associated with better motor execution and coordination.^50-53^ A substantial body of evidence suggests reduced SICI in schizophrenia across different stages of this disorder.^49,54,55^ But the association of this inhibitory impairment with symptoms remains unclear.

Few studies linked SICI to brain imaging measures in schizophrenia.^56-58^ Less inhibition was associated with lower FA in the left corona radiata^57^ and lower rsFC between left M1 and bilateral medial prefrontal cortex, right insula, as well as left cerebellum.^56^ In fact, the effect of white matter properties on SICI was mediated by rsFC in schizophrenia. Still, the contribution of motor symptoms, such as psychomotor slowing, remains unknown.

Given that schizophrenia is associated with (i) alterations in the motor circuitry including higher neural resting-state activity in the sensorimotor cortex; and (ii) lower GABAergic tone and lower intracortical inhibition in the motor cortex, we may speculate that these alterations are most pronounced in patients with current psychomotor slowing, who may present distinct pathobiology.^24^ Thus, this study aimed to test the differences in cortical excitability measures between healthy controls and patients with schizophrenia with or without psychomotor slowing. Furthermore, we probed whether cortical excitability measures were linked to specific alterations of structural and functional brain connectivity in schizophrenia. We hypothesized that SICI was particularly reduced in patients with psychomotor slowing and that corticospinal tract metrics would correlate with MEP amplitudes. Finally, we expected this difference to be pronounced when the patients were classified according to the presence of catatonia, which is associated with extreme forms of psychomotor slowing.^4^

## Materials and methods

### Participants

This cross-sectional study included baseline data of 123 participants from the double-blind, randomized, placebo-controlled trial OCoPS-P (Overcoming Psychomotor Slowing in Psychosis; ClinicalTrials.gov Identifier: NCT03921450). The study was conducted at the University Hospital of Psychiatry and Psychotherapy in Bern, Switzerland between March 2019 and October 2022. Participants included three groups: 60 patients with schizophrenia and psychomotor slowing (PS) according to the Salpêtrière Retardation Rating Scale^59^ (SRRS, total score ≥ 15), 23 patients with schizophrenia without psychomotor slowing (non-PS, SRRS score < 15), and 40 age and gender-matched healthy controls (HC) (Table 1). The study protocol adhered to the Declaration of Helsinki and was approved by the local ethics committee (KEK-BE 2018-02164). Inclusion criteria encompassed being right-handed and aged between 18 and 60 years. In addition, patients needed to fulfil the DSM-5 criteria for a schizophrenia spectrum disorders (more details given in the ‘Clinical characteristics’ section). General exclusion criteria were active substance dependence except for nicotine, neurological disorders impacting motor behaviour, severe brain injury with consecutive loss of consciousness, and contra-indications for magnetic resonance scans and TMS acquisition, i.e. metallic objects in the body or pregnancy. Occasional cannabis consumers were included but were asked to halt consumption in the 24 h prior to MRI and TMS acquisition. Additional exclusion criteria for HC were a history of any psychiatric disorder or any first-degree relative with psychosis. A proportion of this sample was included in reports on grey matter structure in catatonia or structural brain correlates of formal thought disorder.^60,61^

**Table 1:** Demographic and clinical sample characteristics

	HC	PS	Non-PS	Tests	*P*-value
*n* for cortical excitability	40	60	23	–	
Subset for DTI, *n*	38	48	18	–	
Subset for VBM, *n*	40	51	23	–	
Subset for BOLD resting-state, *n*	37	51	23	–	
Age in years, mean ± SD	37 ± 13	37 ± 13	33 ± 12	*F*(2,120) = 0.29	0.746
Sex	19 M	31 M	11 M	χ^2^ (2, *n* = 123) = 3.5	0.740
Education in years, mean ± SD	16 ± 3	13 ± 2	13 ± 2	*F*(2,120) = 22.94	<0.001*
Duration of illness in years, mean ± SD	–	10.7 ± 10.3	8.7 ± 10.7	W = 281	0.650
PANSS Total, mean ± SD	–	81.2 ± 16.9	65.5 ± 14.6	W = 348	<0.001*
PANSS Positive, mean ± SD	–	15.7 ± 5.1	16.3 ± 4.6	W = 763	0.456
PANSS Negative, mean ± SD	–	24.6 ± 6.2	15.9 ± 4.9	W = 176	<0.001*
Antipsychotic OLZ eq. in mg, mean ± SD	–	16.2 ± 11.1	15.1 ± 10.5	W = 653	0.714
Benzodiazepines, *n*	–	13	2	–	
Valproic acid, *n*	–	2	4	–	
Antidepressant, *n*	–	2	1	–	
Lithium, *n*	–	1	0	–	
SRRS	–	23.4 ± 5.6	8.4 ± 2.8	W = 0	<0.001*
BFCRS	–	6.1 ± 4.6	1.3 ± 1.7	W = 157	<0.001*
UPDRS III	–	21.9 ± 12.4	9.0 ± 6.3	W = 237	<0.001*
NES	–	17.1 ± 10.4	10.0 ± 5.6	W = 352	<0.001*
NES motor coordination	–	2.1 ± 2.2	0.6 ± 2.2	W = 329.5	<0.001*

BFCRS = Bush-Francis Catatonia Rating Scale; BOLD = blood oxygen level-dependent; DTI = diffusion tensor imaging; HC = healthy controls; M = male; NES = neurological evaluation scale; non-PS = patients without psychomotor slowing; OLZ eq. = olanzapine equivalent; PANSS = Positive And Negative Syndrome Scale; PS = patients with psychomotor slowing; SD = standard deviation; SRRS = Salpêtrière Retardation Rating Scale; UPDRS III = Unified Parkinson’s Disease Rating Scale Part III; VBM = voxel-based morphometry.

*Significant *P*-value.

### Measures

#### Clinical characteristics

The diagnosis of schizophrenia spectrum disorders (schizophrenia, schizoaffective or schizophreniform disorders) was given according to DSM-5 using the Structured Clinical Interview for DSM-5 (SCID-5) and clinical case files. Symptom severity was measured with the Positive and Negative Syndrome Scale (PANSS).^62^ We assessed motor abnormalities such as psychomotor slowing with SRRS,^59^ catatonia using the Bush-Francis Catatonia Rating Scale (BFCRS)^63^ and the neurological soft signs with the Neurological Evaluation Scale (NES).^64^ All patients were ON antipsychotic medication at the time of testing and mean olanzapine equivalents (OLZ eq.) were calculated according to Leucht *et al*.^65^ Few patients also received benzodiazepines (*n* = 15), antidepressants (*n* = 3), lithium or other mood stabilizers (*n* = 7).

#### Cortical excitability assessment using transcranial magnetic stimulation

The subjects were seated in a comfortable reclining chair during the whole procedure. We used single- and paired-pulse TMS (Magstim Inc.) with a figure-of-eight coil to deliver the stimulations and surface electrodes to record muscle activity (Dantec® Keypoint®). To target the left M1, the standard TMS procedure^66^ is based on the localization of the ‘hand motor hotspot’, i.e. the scalp position where TMS induces the largest MEPs in the first dorsal interosseus muscle. After the hotspot identification, we first determined the resting motor threshold (RMT). The RMT was defined as the minimal stimulus intensity (percentage of the maximal stimulator output) that produced MEPs > 50 µV in peak-to-peak amplitude in at least 5 of 10 trials with the recorded muscle fully relaxed.^66^ Then, we identified the test stimulus threshold (TS), i.e. the minimum stimulus intensity that generated MEPs of 0.5–2.5 mV peak-to-peak amplitude in 5 of 10 trials, i.e. the stimulation intensity was adjusted to obtain stable baseline MEPs of 0.5–2.5 mV. On average, this represented an intensity of 120% RMT. We acquired 15 trials using the TS and averaged the 15 recorded peak-to-peak amplitudes to obtain the MEP amplitude. This measure reflects the general excitability of M1. Finally, we measured the SICI using a paired-pulse TMS paradigm with 1 and 3 ms interstimulus intervals. For each SICI trial, a subthreshold conditioning stimulus (80% RMT) was followed by a suprathreshold stimulus (TS). Owing to the large interindividual variability in cortical inhibition exploration,^67^ we used a standard method^56,58,68^ by acquiring 12 trials for each interstimulus interval and averaged the 24 recorded peak-to-peak amplitudes to obtain the SICI amplitude. Interstimulus intervals of 1 and 3 ms share similar cortical inhibition patterns.^67^

The ratio between SICI and MEP amplitude is used to quantify cortical inhibition. Ratios <1 indicate cortical inhibition; the smaller the ratio, the stronger the cortical inhibition.

#### MRI acquisition procedures

On the same day as the cortical excitability measurements, we performed multimodal neuroimaging at the translational imaging centre of the Swiss Institute for Translational and Entrepreneurial Medicine, Bern, Switzerland. Three neuroimaging markers were acquired: functional connectivity using blood oxygenation level-dependent (BOLD) resting-state functional MRI (rsfMRI), grey matter density using voxel-based morphometry, and structural connectivity using diffusion-weighted images (DWI). The MRI scans were acquired on a 3 T Prisma MRI whole-body scanner using a 20-channel radio-frequency head coil (Siemens). Participants lay horizontally in the magnetic resonance scanner and their arms rested beside their trunk. We placed foam pads around the participants’ head and instructed them to avoid head motion and to not fall asleep.

The MRI protocol encompassed four sequences: a T_1_-weighted MP2RAGE, a field map scan, a diffusion-weighted scan and a BOLD rsfMRI scan (Supplementary material, Section A).

#### MRI processing

##### Diffusion-weighted imaging: structural connectivity

We performed a voxel-wise statistical analysis of FA using tract-based spatial statistics (TBSS) with FSL 6.04 (https://fsl.fmrib.ox.ac.uk/).^69,70^ To evaluate the association of cortical excitability parameters with structural integrity of the motor pathways, we used tractography and computed mean FA for the six main motor-related fibre bundles connecting bilateral SMA, M1 and pathways of motor output [precentral connections via the corpus callosum (CC-precentral); CC-SMA; left and right corticospinal tract (CST), left and right thalamo-precentral connections], using the Quantitative Imaging Toolkit (QIT,^71^https://cabeen.io/qitwiki).

##### BOLD resting-state functional connectivity

The preprocessing of the BOLD resting-state data was similar to the one described in Walther *et al*.^72^ (Supplementary material, Section B). An absolute head motion involving translation >2 mm or rotation >2°, as well as a mean frame-wise displacement (FD) (calculated as defined by Power *et al*.^73^) >0.5 mm were used as exclusion criteria (*n* = 1).

We used the preprocessed rsfMRI data to explore the seed-to-voxel connectivity with a seed (10 mm sphere) created over the left M1 (*x* = −37, *y* = −24, *z* = 56, covering the motor hand knob to reflect the scalp motor hotspot used as the TMS target for the cortical excitability acquisition).

##### Voxel-based morphometry: grey matter density

We used the DARTEL VBM algorithm with SPM 12 (https://www.fil.ion.ucl.ac.uk/spm/software/spm12), following the standard procedure established by Ashburner^74^ to process the grey matter volume and obtained an MRI normalized modulated grey matter smoothing with a 6 mm full-width at half-maximum (FWHM) Gaussian kernel.

### Statistics

The demographic data were compared between groups using ANOVA, Wilcoxon and chi-squared tests. The analyses were performed in three ways: (i) categorical group comparisons [healthy controls (HC), all patients with schizophrenia, psychomotor slowing (PS) and non-psychomotor slowing (non-PS)]; (ii) dimensional associations between MRI measurements and general M1 excitability (MEP amplitude) or cortical inhibition (SICI/MEP amplitude) across the whole sample to explore mechanisms that could be similar irrespective of the clinical status, and within each group to explore mechanisms that are linked to the disorder; and (iii) dimensional associations with measures of psychomotor slowing (a) correlations within all patients with schizophrenia considering the motor abnormalities as a continuum; and (b) correlations within each patient subgroup, i.e. PS and non-PS).

In all analyses, age was used as a covariate. In addition, for analyses focusing on patients only, current antipsychotic dosage (OLZ eq.) was also used as a covariate. Finally, MRI analyses required specific additional covariates depending on the modality. For structural connectivity and grey matter density (GMD) analyses, total intracranial volume was used as an additional covariate whereas for the rsFC analyses, the mean framewise displacement was used. We refrained from comparing the associations of cortical excitability parameters with the neuroimaging markers between groups due to the complex interpretation. Instead, we exclusively performed the associations analyses within each group.

#### Cortical excitability

We used R (https://www.r-project.org/about.html) to perform the cortical excitability analyses. We ran ANCOVAs to compare the cortical excitability measures [RMT, TS, MEP amplitude (general M1 excitability) and ratio SICI/MEP amplitude (cortical inhibition)] between the three groups (PS, non-PS and HC). We used Tukey *post hoc* tests.

To explore the association between cortical inhibition and impaired motor coordination in schizophrenia, we performed a Kendall-tau partial correlation analysis between the ratio SICI/MEP amplitude and an expert rating scale on motor coordination deficits, the NES motor coordination subscore. A *P*-value < 0.05 was considered statistically significant.

#### MRI measurements

##### Structural connectivity

For tractography, we extracted the mean FA values of each of the six fibre bundles and compared them between the groups. We also performed Kendall-tau partial correlations between the FA and cortical excitability measures within each group. A qFDR < 0.05 was considered statistically significant.

In TBSS, we compared the whole brain FA between the groups. We also performed a regression analysis of whole-brain FA with the MEP amplitude as well as the ratio SICI/MEP amplitude within the whole sample as well as within each group (HC, all schizophrenia, PS, non-PS). The level of significance was set at *P*_FWE__corrected_ < 0.05, using a threshold-free cluster enhancement (TFCE) with 5000 randomized permutations.

##### Resting-state functional connectivity

We used the CONN toolbox for seed-to-voxel analysis. Within each group, we explored (i) the rsFC with left M1 as the seed; and (ii) the association between the rsFC seeded on left M1 and the cortical excitability measures: MEP amplitude as well as the ratio SICI/MEP amplitude. We set a cluster-forming threshold of *P* = 0.005 and qFDR < 0.05 for the cluster-wise threshold.

##### Grey matter density

For voxel-based morphometry (VBM) analysis, we used an absolute threshold of 0.1 to ensure the inclusion of grey matter voxels with a probability ≥0.1 of being grey matter. We first compared the whole brain GMD between the groups. Then, to evaluate the association between local GMD and cortical excitability, we used a second-level multiple regression model within each group. We set a cluster-forming threshold of *P* = 0.005 and qFDR < 0.05 for the cluster-wise threshold.

#### Exploratory analyses in catatonia

Finally, we classified all psychosis patients into those with and without catatonia according to the BFCRS screening instrument (scoring at least 1 on two of the first 14 items of the BFCRS).^63^ We repeated analyses with this classification to check whether findings on slowing and catatonia were consistent.

## Results

### Cortical excitability

**Figure 1:**
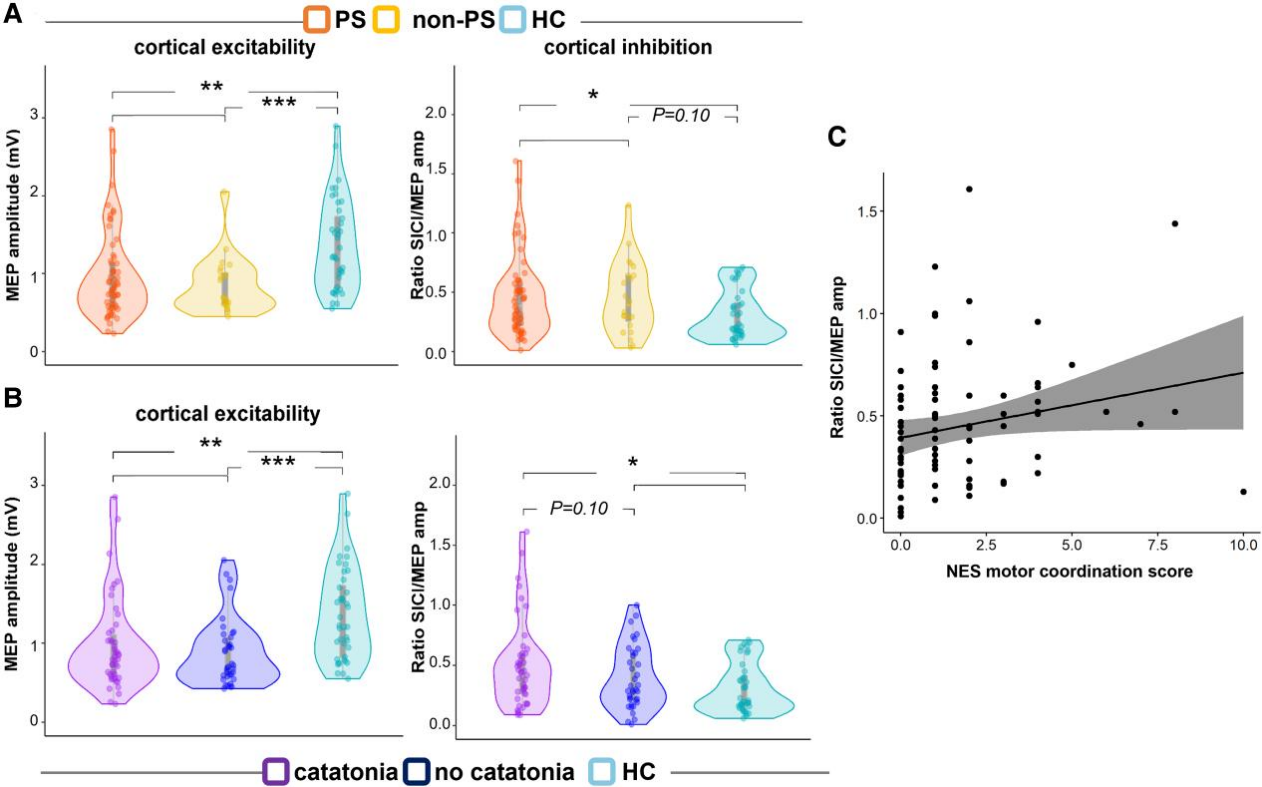
**Cortical excitability measurements related to motor abnormalities.** (**A**) Cortical excitability measurements related to psychomotor slowing. Box and whisker plots of transcranial magnetic stimulation (TMS) threshold (RMT and TS), MEP and ratio SICI/MEP amplitude between groups. (**B**) Cortical excitability measurements related to catatonia. Box and whisker plots of TMS threshold (RMT and TS), MEP and ratio SICI/MEP amplitude between groups. The centre line represents the median value, the lower bound of the box represents the 25th percentile, the upper bound of the box the 75th percentile, and the whiskers represent 3× the interquartile range. *P < 0.05, **P < 0.01, ***P < 0.001. (**C**) Association between cortical inhibition and motor coordination. Correlation plot between the cortical inhibition and the motor coordination subscore of the NES. The solid line is the ‘line of best fit’, a line that minimizes the vertical distances between the data-points and the line itself. The ‘best fit line’ is a useful way of representing the linear trend. The grey shading around the blue line represents the 95% confidence interval around the line of best fit. HC = healthy controls; MEP = motor evoked potential; NES = neurological evaluation scale; non-PS = patients without psychomotor slowing; PS = patients with psychomotor slowing; RMT = resting motor threshold; SICI = short-interval cortical inhibition; TS = test stimulus.

The three groups presented similar RMT (intensity of ∼40%) and TS (intensity of ∼50%) (Table 2). Despite similar motor thresholds, both PS and non-PS patients had reduced MEP amplitudes (i.e. M1 excitability) compared to healthy controls. Likewise, we noted a group difference in cortical inhibition with less inhibition in psychomotor slowing compared to HC. However, there was no difference between the two patient groups in any cortical excitability measure. Results were similar when adding medication as covariate to the model (Fig. 1A). We found no difference in SICI (cortical inhibition) between patients treated with clozapine and those without (Supplementary material, Section C).

**Table 2:** Between-groups comparison of cortical excitability measurements

	RMT (%)	TS (%)	MEP amplitude (µV)	Ratio SICI/MEP amplitude
PS (mean ± SD)	41 ± 7	50 ± 9	967.6 ± 555.4	0.45 ± 0.20
Non-PS (mean ± SD)	42 ± 8	51 ± 9	840.6 ± 358.2	0.45 ± 0.30
HC (mean ± SD)	39 ± 1	47 ± 9	1357 ± 581.9	0.31 ± 0.20
**Main ANCOVA controlling for age**				
Tests	*F*(2,120) = 1.71	*F*(2,120) = 1.62	*F*(2,120) = 9.01	*F*(2,120) = 3.24
*P*-value	0.186	0.203	<0.001*	0.043*
***Post hoc P*-values**				
PS versus HC	0.304	0.328	0.003*	0.048*
Non-PS versus HC	0.201	0.224	<0.001*	0.100
**ANCOVA only in patients only controlling for age and medication**				
Tests	*F*(1,81) = 0.35	*F*(1,81) = 0.28	*F*(1,81) = 1.03	*F*(1,81) = 0.001
*P*-value	0.560	0.600	0.310	0.990

HC = healthy controls; MEP = motor evoked potential; non-PS = patients without psychomotor slowing; PS = patients with psychomotor slowing; RMT = resting motor threshold; SICI = short-interval intracortical inhibition; TS = test stimulus.

*Significant *P*-value.

Across all patients with schizophrenia, we observed a significant positive association between SICI/MEP ratio and the NES motor coordination subscore (tau = 0.15, *P* = 0.047) (Fig. 1C), suggesting more coordination deficits in subjects with lower cortical inhibition. This association was not significant in either of the patient groups separately (PS or non-PS).

### Diffusion tensor imaging

#### Comparison of fractional anisotropy between groups

##### Tractography within bundles

**Figure 2:**
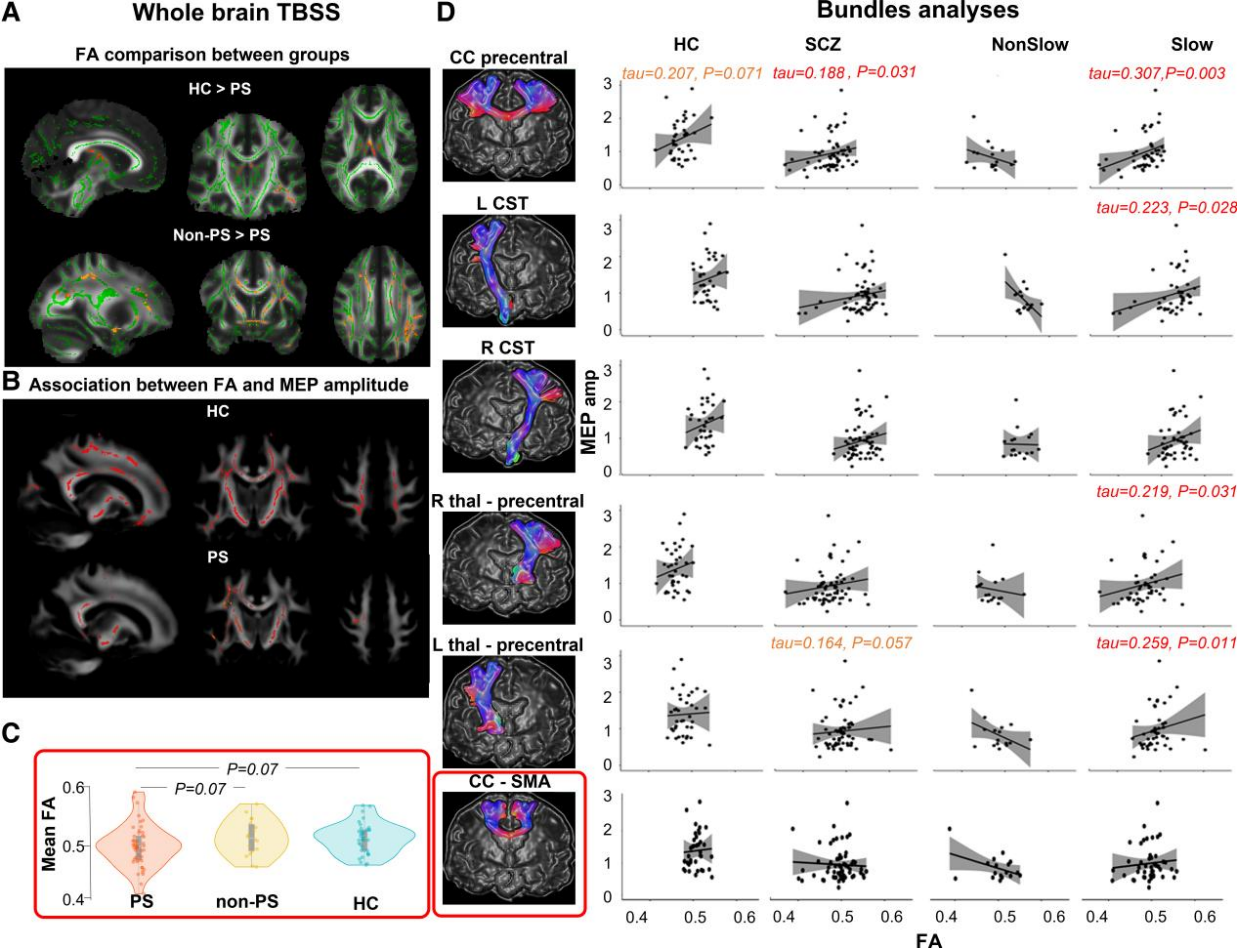
**Association of fractional anisotropy with cortical excitability.** (**A**) whole brain tract-based spatial statistics (TBSS) comparison between groups [healthy controls (HC), patients with psychomotor slowing (PS) and patients without psychomotor slowing (non-PS)]. Clusters in red indicate significant differences between the groups at TFCE *P_FWE_*< 0.05. The fractional anisotropy (FA) skeleton is displayed in green. (**B**) Association between FA (whole brain TBSS) and motor evoked potential (MEP) amplitude. Clusters in red indicate significant positive associations between FA and MEP at *P_FWE_* < 0.05. The FA skeleton is displayed in green. (**C**) Comparison of the mean FA values of the corpus callosum supplementary motor area (CC-SMA) bundles between the groups. (**D**) Association between FA in motor bundles and MEP amplitude within the groups. This figure displays the correlation plots between the MEP amplitude and the FA within the six major motor bundles. In each plot the solid line is the ‘line of best fit’, a line that minimizes the vertical distances between the data-points and the line itself. The best fit line’ is a useful way of representing the linear trend. The grey shading around the blue line represents the 95% confidence interval around the line of best fit. CST = corticospinal tract; FA = fractional anisotropy; HC = healthy controls; L = left; MEP amp = motor evoked potential amplitude; non-PS = patients without psychomotor slowing; PS = patients with psychomotor slowing; R = right; SCZ = schizophrenia patients; SMA = supplementary motor area; thal = thalamus.

The mean FA within the CC-SMA bundle was lower in PS than in both HC and non-PS at trend level (*F =* 2.4, *P =* 0.09; HC-PS: *t =* −2.16, *P =* 0.07; HC-non-PS: *t =* 0.2, *P =* 0.9; PS-non-PS: *t =* −1.9, *P =* 0.07). However, the five other bundles showed similar mean FA between the groups (*F <* 0.3, *P >* 0.5) (Fig. 2C).

##### Whole-brain tract-based spatial statistics

At the whole brain level, PS showed lower FA than HC in the hippocampus area and inferior longitudinal fasciculus. PS also had lower FA than non-PS in the hippocampus area, inferior and superior longitudinal fasciculi, inferior fronto-occipital fasciculus, forceps major and CST. No differences were observed between non-PS and HC (TFCE *P*_FWE_ < 0.05) (Fig. 2A).

#### Association of FA and cortical excitability measurements

No associations between white matter microstructure and cortical inhibition were observed, neither in the TBSS nor the tractography analysis in any group.

##### Tractography within bundles

The tractography across the six fibre bundles showed a significant positive association between FA of the CC-precentral, left CST and bilateral thalamo-precentral tracts and the MEP amplitude across all participants. However, this effect was strongly driven by the PS group (all tau > 0.219, qFDR < 0.031) probably due to the large variation in the FA values within the different bundles compared to the other groups (Fig. 2D).

##### Whole-brain tract-based spatial statistics

At the whole-brain level, the TBSS analyses demonstrated a significant positive association between the MEP amplitude and FA within the sensorimotor pathway (including the CST, frontal aslant tract, the pre/motor, sensory and parietal corpus callosum segments^75^) for all participants, all schizophrenia patients and separately for both PS and HC (TFCE *P*_FWE_ < 0.05). Non-PS did not show significant association between whole brain mean FA and the MEP amplitude (Fig. 2B).

#### Seed-based (left M1) resting-state functional connectivity

##### Comparison of the seed-based resting-state functional connectivity between groups

**Figure 3:**
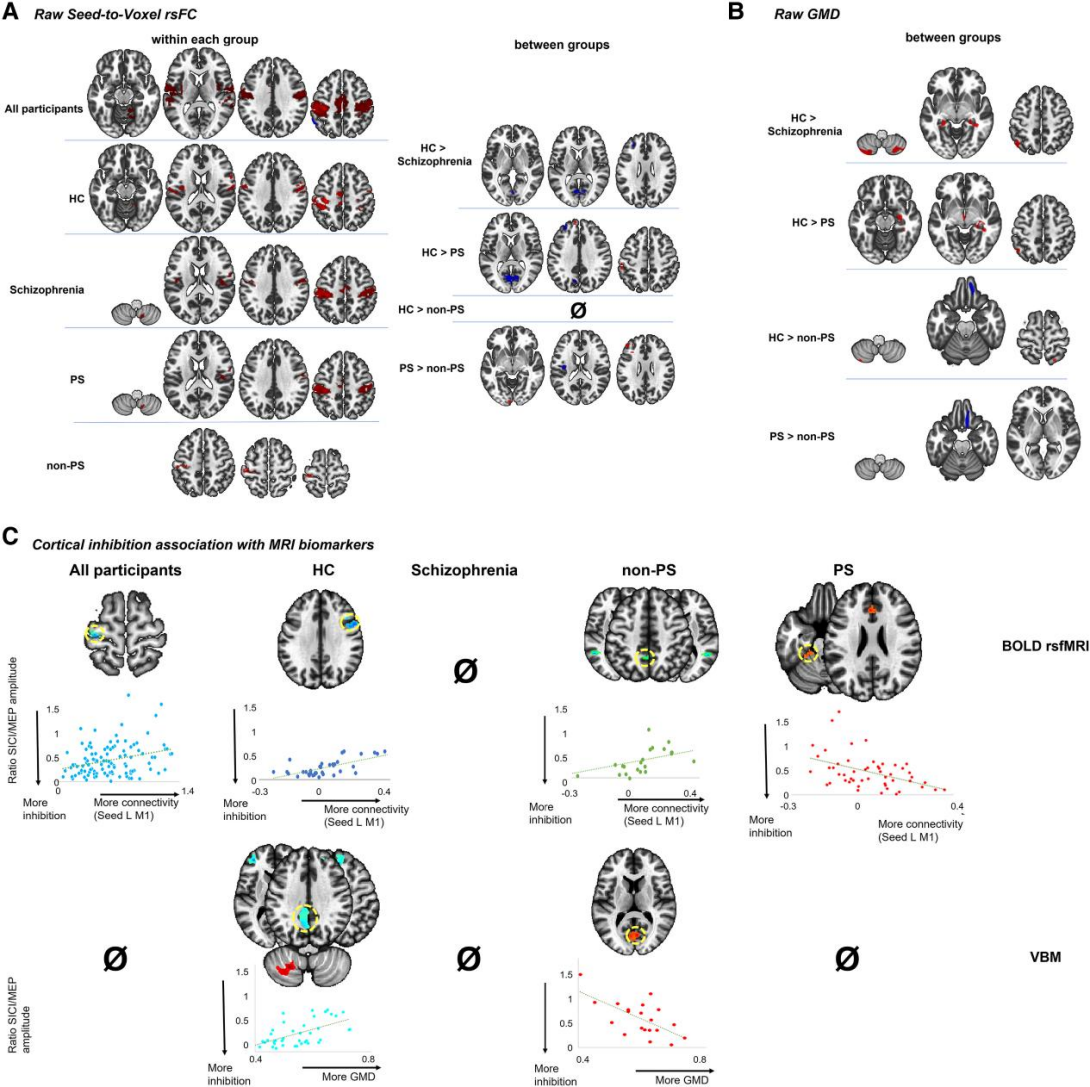
**Association between cortical inhibition and functional connectivity and grey matter density within and between the groups (all participants, controls, all patients with schizophrenia, psychomotor slowing and no psychomotor slowing) cluster-forming threshold of *P* = 0**.**005 and qFDR < 0.05 for the cluster-wise threshold.** (**A**) The seed-to-voxel resting-state functional connectivity (rsFC) seeded on left (L) M1 within and between each group. (**B**) The grey matter density (GMD) within and between each group. (**C**) The association between the SICI/MEP ratio and the strength of the functional connectivity (*top*) or the GMD (*bottom*) within each group. The scatterplots reflect the association between cortical inhibition and the neuroimaging markers within the highlighted (yellow circle) brain area. BOLD = blood oxygen level-dependent; HC = healthy controls; MEP = motor evoked potential; non-PS = patients without psychomotor slowing; PS = patients with psychomotor slowing; SICI = short-interval cortical inhibition; VBM = voxel-based morphometry.

Within all participants, left M1 was highly connected with the other key areas of the motor network (bilateral motor/premotor areas including the SMA and the right cerebellum). This pattern seemed highly preserved across the HC and patients with schizophrenia. However, comparisons between groups revealed connectivity differences between HC and patients with schizophrenia (between left M1 and parietal as well as prefrontal cortices), which is highly driven by PS (Fig. 3A and Table 3).

**Table 3:** Cluster information related to rsfMRI and VBM analyses

Group	Cluster	Size	Cluster *P*_FWE_	Cluster *P*_FDR_	Area
**Functional connectivity between groups**					
Schizophrenia > HC	−08 −70 12	229	<0.001	<0.001	L occipital cortex (BA17)/L parietal cortex (BA 7)
Schizophrenia > HC	−28 40 28	115	<0.010	<0.010	L anterior prefrontal cortex (BA 10)
HC > PS	−14 50 40	107	0.013	<0.010	L DLPFC (BA 9)
	04 36 −20	75	0.100	0.023	R orbitofrontal cortex (BA 11)
	−52 −34 20	64	0.212	0.042	L supramarginal cortex (BA 40)
PS > HC	−06 −70 12	742	<0.001	<0.001	Occipital cortex (BA17)/L parietal cortex (BA 7)
	−28 40 28	265	<0.001	<0.001	L anterior prefrontal cortex (BA 10)
	−30 −40 −32	102	0.018	<0.010	L cerebellum (I, VI)
	−30 −44 −48	59	0.292	0.049	L cerebellum (VIII)
	−02 −46 −18	58	0.312	0.049	L cerebellum (vermis IV–V)
PS > non-PS	−32 34 20	164	<0.001	<0.001	L DLPFC (BA 46)
	−06 −96 −06	77	0.114	0.038	L secondary visual area (BA 18)
Non-PS > PS	−36 −14 16	92	0.045	0.021	L insula (BA 13)
**rsFC association with cortical inhibition**					
More inhibition == lower connectivity					
All participants	−26 −24 62	106	0.014	0.010	L PM (BA 6), L M1 (BA 4)
HC	54 12 40	189	<0.001	<0.001	R PM (BA 6)
Non-PS	−04 −50 48	159	<0.001	<0.001	Precuneous
Non-PS	−52 −54 12	107	<0.010	<0.005	L angular gyrus
Non-PS	48 −60 22	69	0.088	0.037	R angular gyrus
More inhibition == higher connectivity					
PS	−02 28 26	117	0.012	<0.001	L ACC (BA32)
PS	−28 −24 −34	96	0.039	0.016	L cerebellum IV–V
**GMD between groups**					
HC > Schizophrenia	40 −41 −5	4063	0.036	0.016	R hippocampus/R parahippocampus
	−24 −36 −4	3136	0.121	0.034	L hippocampus/L parahippocampus
	26 −69 −57	3254	0.104	0.034	R cerebellum (VIII)
	−44 −70 −57	4229	0.029	0.016	L cerebellum (VIII)
HC > PS	−6 −8 19	3610	0.065	0.028	L caudate/L thalamus/L putamen
	40 −38 −8	4292	0.027	0.028	R hippocampus/R parahippocampus
Non-PS > HC	8 21 −31	3493	0.129	0.041	R orbitofrontal cortex (BA 11)
Non-PS > PS	9 20 −36	6635	<0.010	<0.010	orbitofrontal cortex (BA 11)
**GMD association with cortical inhibition**					
More inhibition == lower GMD					
HC	−10 −57 34	3503	0.020	0.029	L dorsal parietal cortex (BA 31)
HC	1 34 43	3377	0.024	0.029	SMA (BA 6)
More inhibition == higher GMD					
HC	−23 −62 −46	3604	0.020	0.029	L cerebellum VIIIa
Non-PS	−5 −78 3	4133	0.002	0.002	L occipital cortex

ACC = anterior cingulate cortex; BA = Broadmann area; DLPFC = dorsolateral prefrontal cortex; GMD = grey matter density; HC = healthy controls; L = left; M1 = primary motor cortex; non-PS = patients without psychomotor slowing; PM = premotor cortex; PS = patients with psychomotor slowing; R = right; rs = resting-state; rsFC = resting-state functional connectivity; rsfMRI = resting state functional MRI; SMA = supplementary motor area; VBM = voxel-based morphometry.

##### Association of resting-state functional connectivity with cortical excitability measures

We found no associations between the seed-based rsFC and the MEP amplitude in any group. In contrast, across all participants, stronger cortical inhibition was linked to lower rsFC between the left M1 and the left M1/premotor cortex. In HC, more cortical inhibition was associated with lower left M1-right premotor cortex [PM, Broadmann area (BA) 6] rsFC. Interestingly, while in the entire group of patients with schizophrenia a significant association between left M1 and cortical inhibition was lacking, PS and non-PS groups showed an opposing pattern. In PS, stronger cortical inhibition (lower SICI/MEP amplitude) was associated with higher rsFC between left M1-left anterior cingulate cortex (ACC, BA32) and left M1-left cerebellum IV–V. But in non-PS, more inhibition was linked to lower connectivity between left M1 and parietal cortex (bilateral precuneus and angular cortex) (Fig. 3C and Table 3).

#### Grey matter density

##### Comparison of the grey matter density between groups

The between-group differences revealed that HC had higher GMD than schizophrenia patients in bilateral cerebellum (II and VII), bilateral hippocampus, parahippocampus, amygdala, and left parietal cortex. Specifically, the differences in limbic and parietal GMD appeared to be due to decreased GMD in PS. Non-PS showed higher GMD than HC and PS in the orbitofrontal cortex (Fig. 3B and Table 3).

##### Association of grey matter density with cortical excitability measures

MEP amplitudes were unrelated to GMD in all groups. There were no significant associations between cortical inhibition and GMD within all participants or within schizophrenia patients. Conversely, stronger cortical inhibition was associated with lower GMD in the SMA (BA 6) and the left dorsal parietal cortex (BA 31) in HC and with higher GMD in the left cerebellum VIIIa and the left occipital cortex in non-PS. However, no association appeared between GMD and cortical inhibition in PS (Fig. 3C and Table 3).

### Exploratory analysis on catatonia

As an exploratory analysis, we classified the patients into those with and without current catatonia based on the BFCRS (details of these analyses are provided in Supplementary material, Section D). Similar to the results observed with the psychomotor slowing classification, patients with schizophrenia with or without catatonia had smaller MEP amplitudes (reduced excitability) and lower cortical inhibition than HC. Moreover, the patients without catatonia had an intermediate level of cortical inhibition between the controls and the patients with catatonia (Fig. 1B and Table 2). At the rsFC level, the patients without catatonia had similar association patterns as HC (more inhibition associated with decreased left M1-right PM rsFC). However, in the patients with catatonia, more inhibition was associated with increased left M1-SMA and left M1-right cerebellum (vermis VI) rsFC (Supplementary material, Section D).

## Discussion

Motor abnormalities and psychomotor slowing specifically, are frequent symptoms of psychosis but their nature remains widely unknown.^5^ To understand the contribution of motor cortex physiology to psychomotor slowing in psychosis, this study tested cortical excitability and brain connectivity measures in patients with schizophrenia with (PS) and without (non-PS) psychomotor slowing, as well as in HC. We hypothesized that inhibitory deficits would be most pronounced in psychomotor slowing and linked to aberrant connectivity with M1. While TS and RMT were similar across groups, patients had lower MEP amplitudes than HC suggesting reduced general M1 excitability. Furthermore, psychomotor slowing had higher SICI/MEP ratios compared to HC, pointing towards impaired inhibition in psychomotor slowing. This group had distinct neural associations with excitability measures, e.g. lower MEP amplitudes with lower FA in the main motor fibre tracts, while less intracortical inhibition was linked to lower resting-state connectivity between from cerebellum or ACC and left M1. Finally, when patients were classified according to catatonia, results were even more pronounced, suggesting a difference in intracortical inhibition between catatonia and non-catatonia. Furthermore, in catatonia we found lower inhibition to be linked to reduced rsFC between contralateral SMA or cerebellum and left M1. In summary, psychomotor slowing in psychosis is associated with aberrant cortical excitability and inhibition and brain connectivity, suggesting specific pathobiology most likely associated with GABAergic deficits.

Our findings on cortical excitability corroborate and extend prior reports. In line with most studies, we noted no change in RMT in schizophrenia.^49^ Similar to Tang *et al*.,^76^ we found reduced MEP amplitudes in both schizophrenia groups. Furthermore, our MEP results are numerically similar to studies that found no difference from HC.^56^ However, other prior results on MEP have been mixed.^49^ Most previous studies reported reduced SICI or increased SICI/MEP ratios in schizophrenia across all stages of the disorder, indicating impaired intracortical inhibition pointing towards GABAergic deficits.^41,49,54,55^ For our patient groups, SICI/MEP ratios were similar to prior studies,^56,57,77,78^ whereas others found lower ratios in HC. For the first time, impaired inhibition was specifically found in patients with psychomotor slowing compared to HC. PS and non-PS did not differ from each other in SICI/MEP ratios. However, when we stratified patients according to catatonia, patients with catatonia had more pronounced SICI deficits than patients without catatonia at trend level and the difference between catatonia and HC became even clearer. Thus, the TMS data suggest a specific inhibitory deficit in patients with schizophrenia and hypokinetic motor abnormalities, i.e. psychomotor slowing and catatonia.^39,79,80^ This would fit well with the notion of aberrant GABAergic activity in motor cortices in catatonia,^47^ but also to abnormal resting-state hyperactivity in schizophrenia with psychomotor slowing or catatonia.^27,31,33^ Cerebellum, SMA and basal ganglia are known to be part of a complex inhibitory motor network that could cause aberrant inhibition of motor coordination, execution and planning, particularly when this network is driven to hyperactivity.^4,25,81-85^ We may speculate that the dysfunction of GABAergic interneurons may drive tonic cortical hyperactivity in SMA and M1. However, the between-patient comparisons were slightly underpowered and larger samples are required to substantiate this finding. Interestingly, in patients, we detected a correlation between diminished intracortical inhibition and increased coordination deficits (Fig. 1C). Thus, inhibitory dysfunction in M1 was directly linked to impaired motor coordination.

In the group comparisons, PS had lower FA in motor pathways and more rsFC from M1 to prefrontal cortical areas than non-PS. PS also differed from HC in FA and rsFC. Thus, the group not only has distinct behavioural patterns,^24^ but also aberrant neural connectivity in the motor system.^27,86^

One study tested the neuroimaging correlates of intracortical inhibition deficits in schizophrenia, reporting lower inhibition to be associated with reduced FA in the left corona radiata and lower rsFC to the bilateral medial prefrontal cortex, left cerebellum and right insula.^56,57^ In PS, we found similar associations with SICI in resting-state connectivity from left cerebellum lobule VI–V but also the dorsal ACC to left M1: less inhibition indicated lower rsFC. However, this pattern differed clearly from that in HC and non-PS, in whom we detected associations in the opposite direction: less inhibition with increased rsFC to the contralateral PM (HC) or bilateral inferior parietal lobes (non-PS). Thus, normally intracortical inhibition would be facilitated by reduced rsFC from the contralateral premotor cortex, arguing that less information flow from the contralateral premotor cortex would enable M1 to have more intracortical inhibition, which is relevant for a good excitation/inhibition balance. By contrast, patients with psychomotor slowing appear to lack these mechanisms and instead have connectivity patterns from ACC and cerebellum to M1 that seem to restore M1 inhibitory tone by increasing rsFC to M1. Both ACC and cerebellar lobules IV–V have, among other functions, been implicated in movement control^87-89^ and show clear rsFC to M1.^90,91^

In contrast to a previous study,^56,57^ we failed to link SICI to white matter indices. However, this study is the first to find cortical excitability (MEP amplitude) strongly linked to white matter microstructure. This association is somehow expected in the major motor output fibres that transport the signal from left M1 to the muscles. Particularly, linear associations between increased FA and increased MEP amplitude were seen mostly in PS, e.g. in left CST, bilaterally in the tract connecting M1 and thalamus, and in the interhemispheric callosal connection between bilateral M1. The latter was the only pathway in which also HC had a significant association, whereas non-PS had no association at all. In an earlier probabilistic fibre tracking study, we reported that patients had stronger probabilities of structural connectivity between bilateral thalamus and M1 in schizophrenia compared to HC.^26^ Furthermore, in white matter underneath the SMA and M1, we reported higher FA values to correlate with lower physical activity in patients with schizophrenia.^92^ Thus, there is indirect evidence supporting a role for MEP amplitude and motor pathway white matter alterations in aberrant motor behaviour in psychosis.^25^ We found specifically reduced excitability and reduced inhibition in M1 in psychomotor slowing, suggesting a dual dysfunction including glutamatergic and GABAergic neurotransmission in the motor circuits. Previous studies with magnetic resonance spectroscopy found reduced GABA concentrations in occipital cortex and ACC, but not in the basal ganglia.^93,94^ While neuroimaging studies were less clear, post-mortem studies reported reduced GABA in schizophrenia. Furthermore, TMS studies found SICI was reduced in schizophrenia across all stages of the disorder.^45,55^ SICI is thought to reflect to GABA_A_ activity, which in turn is important for behaviour and functional activation in motor cortices.^95^ In fact, SICI reductions in schizophrenia were reported during motor tasks, such as grip maintenance or stop signal inhibition, indicating more noise in the motor system in psychosis.^41,42^ Our findings also align with previous studies demonstrating a link between neurological soft signs in psychosis and increased cerebello-frontal rsFC,^96^ as well as glutamatergic dysfunction.^97^

In summary, our findings add to the growing literature on excitation/inhibition imbalance in schizophrenia,^40^ suggesting a role in aberrant motor behaviour.

The present study included a moderately sized group of severely ill patients for close clinical phenotyping, TMS experiments and MRI. Experiments were conducted on the same day as the MRI scans with consistent timing across the entire sample. However, some limitations of the current study require discussion. First, the non-PS group was comparably smaller than the PS and control groups, which may have led to lower statistical power for the group comparisons. Second, our patients were mostly ill and thus receiving medication. We controlled for medication dosage but cannot rule out any impact of medication on either cortical excitability measures or MRI parameters. However, we could not control for cumulated lifetime antipsychotic exposure or for the duration of the current medication. Still, SICI measures may be influenced more by symptoms than by medication.^49^ The lack of significant difference between the PS and non-PS groups for the cortical excitability measurement could be explained by the aforementioned reasons or variability of scores in the expert ratings. Future studies need to be performed using larger samples for both patient groups and classification methods based on instrumental measures to provide a better overview. During the MRI acquisition, the participants were only asked to avoid motion and sleep. A careful monitoring as well as a precise instruction might have reduced intra-individual variability in the state of mind during the resting-state scans. Finally, our HC were closely matched for age and sex to our patient groups but presented slightly higher SICI/MEP ratios and MEP amplitudes than in some of the previous literature.

In summary, this study demonstrates reduced excitability and diminished intracortical inhibition in the motor cortex of patients with schizophrenia and psychomotor slowing. The inhibition deficit is linked to poor motor coordination and altered rsFC in the motor circuit. This excitation/inhibition imbalance within the motor circuitry might give rise to psychomotor abnormalities in psychoses.

## Supplementary Material

awad395_Supplementary_Data

## Data Availability

The dataset presented in this article is not readily available because some participants did not provide consent to data sharing. Requests to access the datasets should be directed to S.L., stephanie.lefebvre@unibe.ch.
