# Supplementary material for: The pathobiology of psychomotor slowing in psychosis: altered cortical excitability and connectivity
Source: Brain. 2023 Nov 20;147(4):1423–35. doi: 10.1093/brain/awad395 (PMC10994557; doi:10.1093/brain/awad395)
Supplement: awad395_Supplementary_Data [file awad395_supplementary_data.pdf]

The mechanism of psychomotor slowing in psychosis: altered cortical excitability and connectivity

Stephanie Lefebvre,Niluja Nadesalingam, Melanie G Nuoffer, Alexandra Kyrou, Florian Wüthrich, Sebastian Walther

Supplementary Material

Table of contents

A. MRI acquisition procedures

B. rsFC processing

C. Medication effect on SICI

D. Exploratory analysis on Catatonia

A. MRI acquisition procedures

The MRI protocol included the following 4 sequences:

1. A structural scan: T1-weighted MP2RAGE images (8 minutes 22 seconds covering 176 sagittal slices, 1 mm thick, TR = 5000 ms, TE = 2.98 ms, flip angle 1= 4°, flip angle 2= 5°, voxel size = 1x1x1 mm).
2. A field map scan for use in unwarping DWI distortions due to magnetic field inhomogeneity: FOV = 217×217 mm, 72 slices, TR = 707 ms, TE1 = 4,92 ms, TE2 = 7.38 ms, Flip angle = 60°, thickness = 2.5 mm, Voxel size = 2.4 *2.4*2.5mm.
3. A diffusion-weighted MRI scan with echoplanar images of 123 directions in antero-posterior encoding direction (TR=3700 ms, TE=82 ms, FOV 211 mm × 211 mm, slices = 56, thickness = 2.2 mm, PAT = 4, multi-shell acquisition with b0 and 8 different b-

values : b=0, b=350, b=650, b=1000, b=1350, b=1650, b=2000, b=2650, b=3000 s/mm^2 acquisition duration = 7.55 min, Voxel size = 2.2*2.2*2.2 mm.

4. A BOLD rs-fMRI scan using a multi-band echoplanar 2D (10 minutes and 11 seconds covering 600 volumes of 72 slices; 2.5 mm thick, TR =1000 ms, TE = 37 ms, flip angle =30 °, voxel size = 2.5×2.5×2.5 mm FOV = 230x230 mm, GRAPPA =1, multiband acceleration factor = 8).

B. rsFC processing

We processed the rs-fMRI data using SPM12 to slice-time correct, motion realign, co-register and normalize the functional scans. We performed the denoising steps with the CONN toolbox v20 (functional connectivity toolbox; http://www.nitrc.org/projects/conn) using noise components of white matter and cerebrospinal fluid tissues and motion parameters (6 motion parameters and their associated first-order derivatives), a despiking procedure, a linear detrending and band-pass filtering (0.01–0.1 Hz).

C. Medication effect on SICI

C1. Effect of OLZ

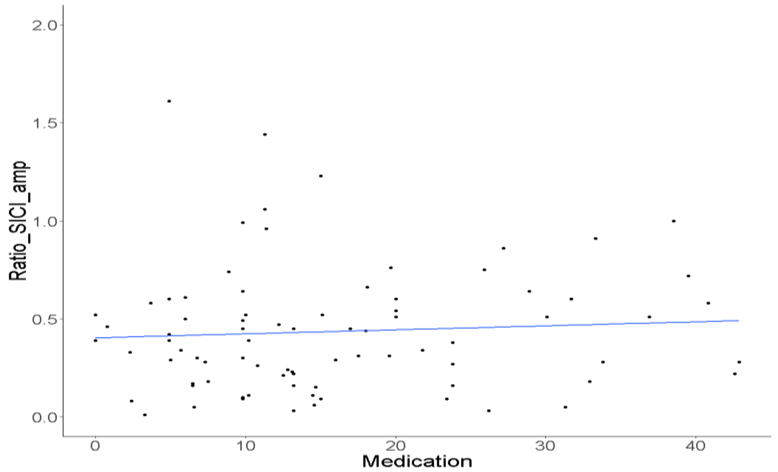

P value	tau
0.5084	0.05063

Correlation plot between Medication (OLZ) and the cortical inhibition measurements.

C2. Effect of clozapine

Only 18 patients used clozapine medication at the time of the study, all in the PS group. A Wilcoxon test demonstrated no difference in SICI between the patients treated with and without clozapine (W = 446.5, p-value = 0.27)

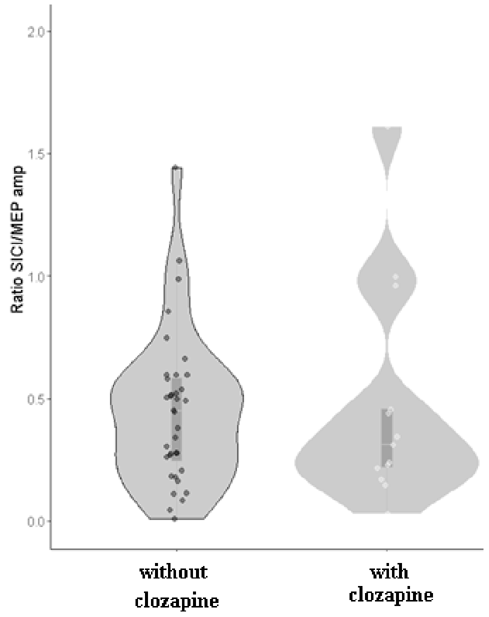

Clozapine	N	Ratio SICI/MEP amp	sd	se	ci
without	42	0.4593	0.3098	0.04781	0.09655
with	18	0.4222	0.3984	0.0939	0.1981

D. Exploratory analysis on Catatonia

	HC	C	NC	Group comparison (catatonia)
N (for cortical excitability)	40	46	37	
		44 with PS	16 with PS	
Age in years (mean ± sd)	37 ± 13	37 ± 13	35 ± 12	F (2, 120) = .65, p = 0.520
Sex	19 M	24 M	18 M	X^2 (2, N = 123) = 5.49, p = 0.484
Education in years (mean ± sd)	16 ± 3	12 ± 2	12 ± 2	F (2, 120) = 23.01, p < 0.001*
Duration of illness in years (mean ± sd)	-	9.5 ± 10.5	11.5 ± 9.6	W = 622, p = 0.15
PANSS Total (mean ± sd)	-	84.1 ± 17.2	67.8 ± 13.8	W= 406, p < 0.001*
PANSS Positive (mean ± sd)	-	15.7 ± 4.6	15.9 ± 5.2	W= 862, p = .919
PANSS Negative (mean ± sd)	-	25.7 ± 6.3	17.7 ± 6.2	W= 264, p < 0.001*
Medication OLZ eq. in mg (mean ± sd)	-	15.4 ± 9.8	16.4 ± 12.3	W = 841, p = .931
BFCRS	-	7.4 ± 4.5	1.6 ± 1.6	W= 117, p < 0.0001*
NES	-	17.4 ± 10.6	12.5 ± 8.1	W= 543, p = 0.005*
NES motor coordination	-	2.2 ± 2.3	1.1 ± 1.4	W= 559, p = 0.005*

HC: Healthy controls; C: Catatonia; NC: no catatonia

Demographics and clinical information in Catatonia classification

To be classified into the with catatonia group, patients need to score at least 1 in two of the first 14 items of the BFCRS scale.

Catatonia cortical excitability results

	RMT	TS	MEP amplitude	Ratio SICI/MEP amplitude
**HC (mean ± sd)**	39 ± 1	47 ± 9	1357 ± 581.9	0.31 ± .20
**C (mean ± sd)**	41 ± 8	51 ± 9	957.6 ± 541.8	0.50 ± 0.35
**NC (mean ± sd)**	42 ± 8	50 ± 9	907.7 ± 957.6	0.39 ± 0.25
**Main ANCOVA (controlling for age)**	$F(2, 120) = 1.71$, $p = 0.186$	$F(2, 120) = 1.5$, $p = 0.223$	$F(2, 120) = 8.58$, $p < 0.001$*	$F(2, 120) = 4.79$, $p = 0.09$*
**Post Hocs**	C vs HC: p = 0.399<br>NC vs HC: p = 0.179	C vs HC: : p = 0.394<br>NC vs HC: p = 0.221	C vs HC: : p = 0.003*<br>NC vs HC: p < 0.001*	C vs HC: p < 0.01*<br>NC vs HC: p = 0.28
**ANCOVA patients (controlling for age and medication)**	$F(1, 81) = 0.22$, $p = .64$	$F(1, 81) = 0.09$, $p = 0.76$	$F(1, 81) = 0.19$, $p = 0.66$	$F(1, 81) = 2.407$, $p = 0.10$

HC: Healthy controls; C: Catatonia; NC: no catatonia

Cortical inhibition associated with Seed-based connectivity (L M1) in with and without patients with catatonia

	Cluster	size	cluster p FWE	cluster p FDR	area
**Catatonia**	More inhibition == increased connectivity				
	08 14 62	117	0.035	0.036	SMA (BA 6)
	02 -78 -14	101	0.079	0.038	R cerebellum (Vermis VI)
**Without catatonia**	*More inhibition == decreased connectivity*				
	54 12 40	189	< 0.001	< 0.001	R PM (BA 6)

Non Catatonia **Catatonia**

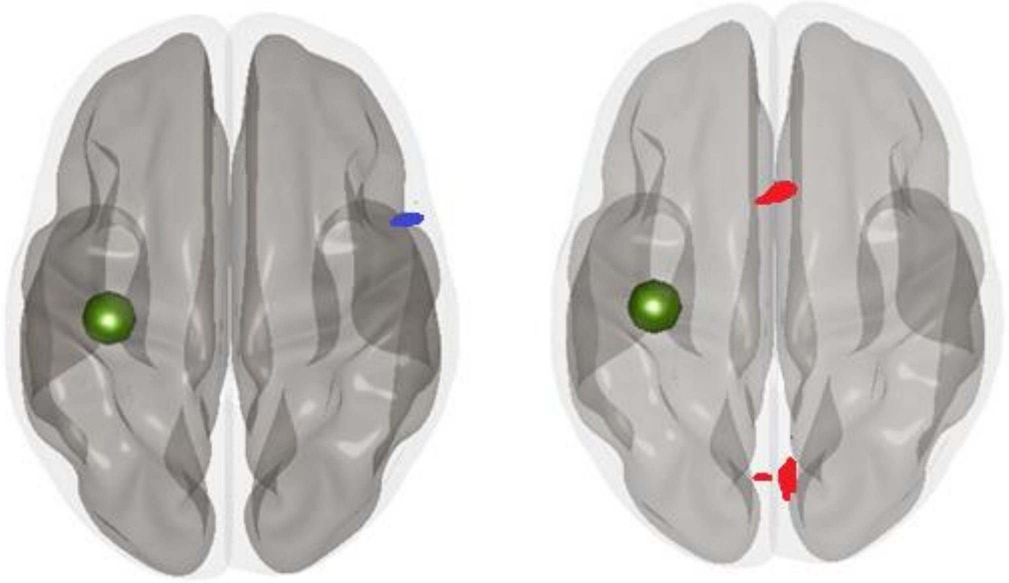

More inhibition => decreased connectivity between L M1 and R PM

More inhibition => increased connectivity between L M1 and R SMA and R cerebellum Vermis VI

Association between functional connectivity and Cortical inhibition
